# Digital Transformation in Materials Science: A Paradigm Change in Material's Development

**DOI:** 10.1002/adma.202004940

**Published:** 2021-01-06

**Authors:** Julian Kimmig, Stefan Zechel, Ulrich S. Schubert

**Affiliations:** ^1^ Laboratory of Organic and Macromolecular Chemistry (IOMC) Friedrich Schiller University Jena Humboldtstr. 10 Jena 07743 Germany; ^2^ Jena Center for Soft Matter (JCSM) Friedrich Schiller University Jena Philosophenweg 7 Jena 07743 Germany

**Keywords:** artificial intelligence, automation, combinatorial science, digital transformation, machine learning

## Abstract

The ongoing digitalization is rapidly changing and will further revolutionize all parts of life. This statement is currently omnipresent in the media as well as in the scientific community; however, the exact consequences of the proceeding digitalization for the field of materials science in general and the way research will be performed in the future are still unclear. There are first promising examples featuring the potential to change discovery and development approaches toward new materials. Nevertheless, a wide range of open questions have to be solved in order to enable the so‐called digital‐supported material research. The current state‐of‐the‐art, the present and future challenges, as well as the resulting perspectives for materials science are described.

## Graphical abstract

The ongoing expansion of digitalization approaches influences material research significantly. The complete workflow of the development of novel materials, from synthesis, over characterization, to fabrication will change within the coming years to a more automatic, data‐driven, and robot‐based approach. The current status is summarized and advantages, challenges, and future perspectives discussed.

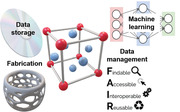

## 1 Introduction

Artificial intelligence (AI), automation, deep learning, big data, machine learning: these buzzwords are dominating the media, in news articles and are already connected to nearly every field and application (see **Table** **1** for definition of the most important terms). Consequently, it is not surprising that science in general is not excluded from this trend and there are already first promising results.^[^ ^1^ ^]^ Exemplarily, a deep learning software can be utilized for the identification of specific genetic diseases,^[^ ^2^ ^]^ artificial intelligence can be utilized for the interpretation of images in clinical routines^[^ ^3^, ^4^ ^]^ or the development of new drugs,^[^ ^5^ ^]^ big data handling simplifies the understanding of geographic questions^[^ ^6^ ^]^ or cognitive computing enhances the speed of life science research.^[^ ^7^ ^]^ All these examples show the great potential of the ongoing digitalization for significant advancements in science and one can simply conclude that this process will also influence materials science and the way research will be performed in the future. Thus, here, we will focus on the current developments in terms of automation and digitalization in all fields of material research (covering all material classes), evaluate the current and future challenges, and try to draw a picture of a potential digital material research future, which is beginning to develop. For this purpose, we will focus on the different aspects of material development starting from the experiment itself over the characterization and analysis of the data and finally to the fabrication and application of the obtained materials.

**Table 1:** Definition of often‐utilized terms for the field of automation and digitalization

Term	Short definition^a)^
Artificial intelligence (AI)	“The theory and development of computer systems able to perform tasks normally requiring human intelligence, such as visual perception, speech recognition, decision‐making, and translation between languages.”
Machine learning	“The use and development of computer systems that are able to learn and adapt without following explicit instructions, by using algorithms and statistical models to analyze and draw inferences from patterns in data.”
Big data	“Extremely large data sets that may be analyzed computationally to reveal patterns, trends, and associations, especially relating to human behavior and interactions.”
Deep learning	“A type of machine learning based on artificial neural networks in which multiple layers of processing are used to extract progressively higher level features from data.”

^a)^ Definitions were obtained from English Oxford Living Dictionary.

## 2 Preparation of Materials

The first and one of the most important steps in the development of new materials is the synthesis, formulation or preparation of new compounds. Typically, this step marks the beginning of material development and is followed by characterization (after a potential purification) and interpretation of the obtained data.

In general, new materials are obtained by a chemical reaction, a modification or by formulation/blending of different substances. The reaction conditions must be chosen accordingly to obtain the material of choice with the appropriate properties. Typically in today's research, these syntheses are performed by human, that is, scientists or technicians, who utilize a wide range of different methods for the different classes of materials. While in the field of polymers mostly classical organic synthesis protocols are utilized,^[^ ^8^ ^]^ there are also formulation such as sintering steps (e.g., for ceramics),^[^ ^9^ ^]^ melting processes,^[^ ^10^ ^]^ or sol–gel processes^[^ ^11^ ^]^ for other material classes, for example, metals, respectively.

This approach has the great disadvantage that the results and the materials obtained depend strongly on the researcher performing the experiments and on how much experience one has. For this reason, initial approaches in the digital transformation of material research are focusing mostly on the automation, parallelization, and miniaturization of the synthesis and the development of opportunities for high‐throughput processes.^[^ ^12^ ^]^ Two priority methods are robot‐based synthesis^[^ ^13^ ^]^ and flow chemistry^[^ ^14^ ^]^ (including microfluidics^[^ ^15^ ^]^). The former one is based on the utilization of synthesis robots, which can perform a variety of experiments simultaneously and with high precision as well as high reproducibility. In contrast, in flow chemistry microreactors, for instance, are utilized allowing a corresponding high number of experiments due to the parallel experiments as well as a relatively low amount of required compounds.^[^ ^16^, ^17^, ^18^ ^]^ Both approaches can also be combined as shown in a very recent study.^[^ ^19^ ^]^ Herein, a robotic arm is utilized to enable flow chemistry approaches. Additionally, the system features an AI‐software enabling an improvement in the experimental planning. However, this system can only be utilized for organic synthesis protocols and cannot easily be adapted for other material classes such as metals or ceramics.

These two approaches have been known for a while,^[^ ^8^ ^]^ but they have progressed differently in the different material classes. Consequently, synthetic robots are well‐known in the field of polymer synthesis as well as polymer formulation and have been used for a variety of processes.^[^ ^20^, ^21^ ^]^ However, most of the syntheses performed nowadays are still classical ones using flasks and other classical laboratory glassware. In addition, catalysis represents a research field with a strong utilization of combinatorial approaches.^[^ ^22^ ^]^ Also in other material classes there are first examples in this direction,^[^ ^23^ ^]^ for example, in the perovskite development^[^ ^24^ ^]^ or alloys,^[^ ^25^ ^]^ nevertheless, the automated synthesis/formulation has not yet been fully enforced and many syntheses and material manufacturing processes are still performed in a classical manner. This is also due to one of the disadvantages of the robotic approach, which are high costs of the required equipment. However, this investment would be a one‐time investment, which can potentially be cheaper on a long‐term perspective compared to paying scientist.

An increasing trend toward automated synthesis is recognizable, since it not only increases the number of experiments, it further generates more reliable data not influenced by human bias. Each experiment is traceable, the reaction conditions can be read out as a protocol (this data is thus usable, see paragraph data management) and a new experiment under the same conditions provides comparable results (also in different labs as long as the same starting materials are utilized). Therefore, it is not surprising that companies have recognized this trend and have heavily invested in the automation of syntheses/formulation for their research and development departments.^[^ ^26^, ^27^ ^]^

The challenges in the field of automation and digitalization in the area of material preparation are to be sought above all in the improvements of the robotic systems and processes. In particular, the robotic systems have to be optimized to cover all different reaction conditions, which are applied in material research (temperature, pressure, type of material etc.). Exemplarily, high‐temperature processes have to be mentioned, which is still a challenge using automated synthesis robots. Moreover, the required compounds (liquids, solids, pastes, oils etc.) have to be transferred from bottles, boxes, cylinders etc. into the reaction vial in the right volume, mass using syringes or balances. A novel revolutionary approach using “pins” (or “coins”) has been presented in order to overcome the this bottleneck of current robotic based synthesis.^[^ ^28^ ^]^

Furthermore, multi‐step reaction procedures have to be performed automatically.^[^ ^29^ ^]^ In addition, the purification and characterization of the materials must be performed in such an automated fashion. First approaches to this are well‐known in the field of organic synthesis and polymer science.^[^ ^30^ ^]^ Thus, the group of Cronin has combined various purification and analysis methods with synthesis modules.^[^ ^31^, ^32^ ^]^ Transferring this approach to the various material classes and characterization methods will be a crucial challenge for the future of the digital transformation of material research.

**Figure 1:**
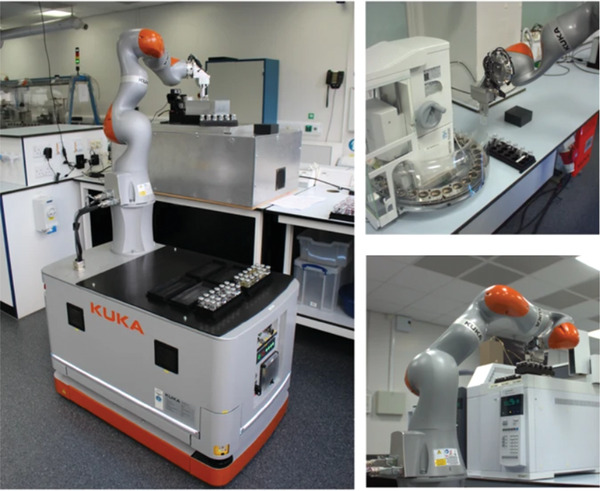
A robotic mobile chemist at work filling vials and loading the gas chromatography equipment. Reproduced with permission.^[^ ^33^ ^]^ Copyright 2020, Springer Nature.

Recently, a synthetic robot, which can move through a laboratory collecting samples, placing them into analysis equipment, such as gas chromatography or high‐performance liquid chromatography, was described by the Cooper group (**Figure** **1**).^[^ ^33^ ^]^ Herein, the robot was improved using a learning algorithm in order to search for photocatalysts. For this purpose, the robot performed more than 600 experiments and was able to find a better working catalyst compared to the initial starting material by using a learning algorithm.

## 3 Characterization of Materials

The characterization and analysis of materials have been performed already for a very long time using automated equipment, for example, with auto samplers, well plates, etc. Nonetheless, there is an urgent need for further development in order to facilitate effective digitalization and automation, since the characterization represents a central element for the generation of the (measurement) data.^[^ ^34^ ^]^ For this purpose, also additional on‐demand and online approaches are required. A major disadvantage for a uniform procedure across all material classes in the development of the analysis methods is the great variety of the materials, the investigated properties as well as the techniques themselves (provided by different companies and utilizing different software tools). Here, even within one class of material, for example, polymers, one can find a wide range of techniques that are regularly applied and used. This challenge multiplies enormously across the different material classes and makes increasing automation and digitalization even more difficult with increasing networking between the devices.

Generally, the requirements profile of analytical methods changes to a faster characterization at a constant quality standard, enabling, for example, direct real‐time (kinetic) investigations. This is usually associated with the term of high‐throughput characterization in which a variety of samples can be analyzed within a short timeframe (also online and inline), which are known for different material classes, for example, polymers, alloys or hydrogels.^[^ ^35^, ^36^, ^37^ ^]^ The increasing need for faster characterization is also due to the increasing amount of data required for machine‐learning algorithms, as they can only work effectively if sufficient data is generated and implemented.^[^ ^38^ ^]^ The increasing automation of synthesis will increase the need for new (faster) characterization approaches.

Nevertheless, the general procedure for the characterization of new compounds applied so far consists of the sample collection during synthesis and the subsequent transfer to the characterization device by hand. Afterward, the measurement data are evaluated independently by the scientist. However, this procedure must be optimized so that a more efficient and faster analysis is possible and bottlenecks in the research can be eliminated. Variants for this are, for example, the online characterization during the reaction,^[^ ^39^ ^]^ the combination of synthesis, purification, and characterization (see earlier)^[^ ^31^, ^32^, ^40^ ^]^ and online/inline flow‐characterization approaches.^[^ ^41^ ^]^ All of these strategies are already available to a certain extent, however, they are rarely utilized, (combined) expensive and need to further be developed.

Finally, another important strategy in the field of materials analysis is the automatic evaluation of the obtained results. Mostly nowadays the results are evaluated by hand by the scientist. To optimize this process, increasingly automatic evaluation tools are applied. Exemplarily, the machine‐based image recognition (e.g., transmission electron microscopy analysis)^[^ ^42^ ^]^ or the automatic analysis of spectra (e.g., nuclear magnetic resonance)^[^ ^41^ ^]^ have to be named.

In particular, the aspect of the accuracy of the analysis should not be lost during all automation digitalization and time‐related improvements and the obtained information should not change during improvements. Only trustworthy measurement data can be utilized by machine learning and artificial intelligence. If this would not be the case, such programs would predict new experiments and properties based on wrong data resulting in incorrect predications.

## 4 Management and Analysis of Data

The collection, analysis and handling as well as the interpretation of data represents the central element in the field of digitalization in material research. However, these aspects have received the least attention so far. There are frequently reports on the utilization of artificial intelligence etc.,^[^ ^1^, ^38^, ^43^ ^]^ but very little on the storage and (automated) interpretation of data leading to new knowledge and an accelerated materials discovery. However, they are the key elements, as shown, for example, by Amazon and others for their purposes, but not yet for materials science. The data are required to train software programs in the field of machine learning and artificial intelligence enabling automated routines to provide personalized solutions and suggestions. A first initiative that has recognized the potential in this field was the material genome project of the US government^[^ ^44^ ^]^ and later the analog initiative in China.^[^ ^45^ ^]^ In doing so, the competencies in the area of material research should be bundled and by a combined effort it should finally be possible to decrypt the “material code”^[^ ^1^ ^]^ opening the field of material informatics, “materialomics” or “polymeromics.”^[^ ^46^ ^]^

Currently, most of the research data is generated locally in academic and industrial laboratories and only there the primary data are accessible, stored in different formats at different storage location, which are partially offline. Furthermore, even in the corresponding scientific publications only selected data are presented, typically only the data from successful experiments.^[^ ^47^ ^]^ Thus, only very sparse, the really relevant data are shared within the scientific community. The quality of the provided data is sometimes questionable and the reliability is not given in every case. Another challenge in this context is the “negative” data, which are data obtained from experiments not resulting in the desired material.^[^ ^48^ ^]^ These data are hardly published these days,^[^ ^47^ ^]^ or even stored in a useful manner. Of course, this raises the question of how many experiments have already been carried out meaninglessly several times in the world, even though it has already been observed that the experiments are not promising.

In order to merge all data, positive and negative ones, a comprehensive database structure for all data of the material research is required. Meta‐analysis, as known from other disciplines,^[^ ^49^ ^]^ are nowadays hardly possible and higher‐level structure‐property relationships can only be revealed with great difficulty. In addition, the design and planning of new experiments are not optimal.

One solution to this problem is provided by the FAIR principle of data handling.^[^ ^50^ ^]^ Thus, the data are findable, accessible, interoperable and reusable. Consequently, previously used experiments and the associated data can also be reutilized again. A central element for storing the data is required, which can be offered by a repository. All original data are stored in a meaningful way applying the FAIR principles. Consequently, all these data can be (re)used. Finally, new results can be generated based on existing measurements, and meaningless experiments are not performed multiple times. Moreover, less promising procedures can be later improved in an automated manner. Such an example can be found in the field of metabolomics and is already known and established.^[^ ^51^ ^]^ Herein, the measurement data in a specific format are stored centrally in a public repository and each scientist around the world can access and reuse those data. One approach regarding materials science is the material cloud,^[^ ^52^ ^]^ for example, Aflowlib with over 3 000 000 million substances.^[^ ^53^ ^]^ One current positive trend is that more and more journals already starts to expect the storage of primary data related to a publication. However, this procedure must be improved in the future and the data storage has to become obligatory for each publication.

Nevertheless, there will be further challenges^[^ ^54^, ^55^ ^]^ in the form of generally accessible data formats, the standardization of data and, consequently, the definition of information standards. Moreover, also already obtained knowledge must be converted into data formats in order to make them usable. As a basic requirement, scientists themselves must be convinced of such a system and students have to be trained accordingly. A first national step is currently being observed in Germany, where a national research database infrastructure is to be set up (e.g., the initiative NFDI4Chem^[^ ^56^ ^]^ for chemistry related fields). However, global solutions are required. Moreover, data storage and management work flows must be generated, which are useable for all material classes.^[^ ^57^ ^]^

## 5 Planning of Experiment/Design of Experiments

The category of experimentation planning is, for most people today, most associated with the concept of digitalization. Software‐assisted synthesis planning and the development of new materials using machine‐learning and artificial‐intelligence approaches can become a key technology in the future of materials research.^[^ ^43^ ^]^

Classically, the planning of experiments is performed in such a manner that the scientist considers a new experiment based on literature findings and personal experience. This experiment is then performed and, based on the obtained data, a new experiment may be planned.

The first advances in this field are provided by simulations of reactions and material properties that allow a prediction of particular compositions and, thus, enable more targeted experiments.^[^ ^58^ ^]^ In the first phase of the development of such simulations, the results were still rudimentary and could only partially be utilized for the prediction of properties. Meanwhile, the prediction quality have become much better (also using machine‐learning techniques^[^ ^59^ ^]^) and simulations allow in certain areas, for example, metal–organic frameworks (MOFs),^[^ ^60^ ^]^ the targeted development of new materials.^[^ ^61^, ^62^, ^63^ ^]^ The simulation of properties and the corresponding structures becomes increasingly more important; however, it is still limited for specific material classes such as alloys^[^ ^64^ ^]^ or MOFs.^[^ ^65^ ^]^ In case of, for example, polymers, the modeling is still in its infancy and the calculation demand is too large to obtain exact predication.^[^ ^66^ ^]^ In the future, simulations will gain more importance in all fields of materials and can also be utilized for the achievement of data, which can be further used for artificial intelligence programs.

The next step in a modern material research process is the utilization of machine learning and artificial intelligence to facilitate feedback‐driven reaction and material planning.^[^ ^38^, ^43^ ^]^ These programs uses the data from previous experiments predicting a new targeted composition of a material, which can subsequently be synthesized. Alternatively, the measurement data may also be utilized for the improvement of the synthesis protocol and, consequently, the yield of the reaction. Finally, a sufficient amount of data can be generated by a large number of experiments resulting in the opportunity to reveal the ideal composition of a material for a certain application. Above all, it is important that sufficient data points are available (as the number of desired properties increases, the number of data required also increases) and that negative data attempts are also recorded,^[^ ^67^ ^]^ since otherwise the desired parameter space cannot be defined in a clear manner.^[^ ^38^ ^]^

The development of such software‐based methods for material development of new materials is still in its infancy. However, there are first examples, such as the use of machine‐learning for the predication of material properties,^[^ ^59^, ^68^ ^],^ for example, battery materials,^[^ ^69^, ^70^ ^]^ the use of machine learning and modeling for the targeted selection and subsequent synthesis of conductive MOFs,^[^ ^71^, ^72^ ^]^ the predication of metallic glasses^[^ ^73^ ^]^ or the targeted development of catalysts.^[^ ^57^ ^]^

The most significant challenge for machine‐learning systems is the amount of data and the associated time (and resources) for the required experiments. The better the program should work and the more properties a particular material has, the more experiments are required. To solve this challenge, there are two possible solutions: a) the utilization of simulations^[^ ^74^ ^]^ to predict material properties^[^ ^75^ ^]^ (as well as the utilization system properties and similarities^[^ ^76^ ^]^) and b) the merging of all generated data in a corresponding repository from which the software can obtain the required data. This procedure could reduce the number of required experiments and enable a more targeted material research in the future. However, at the present time, the application of simulation is limited to some material classes and a general (worldwide used) repository for materials research containing all classes of materials is not foreseeable. Accordingly, the current descriptions of machine‐learning‐based material systems are mostly based on the use of own data (partially evaluating the literature) and, thus, demonstrate only the principle feasibility of such systems.

## 6 Fabrication and Processing

The processing and fabrication of materials represent usually the final step in the exploration of future potential applications of the materials. The way of processing largely depends on the materials properties and the potential fields of application, whether the usage is, for example, for batteries, as a coating for anticorrosion or as construction element. Here, a variety of different techniques are already available, which can also run robotically supported, already (partially) applied industrially. The classic processes here are, besides many others, extruding, spraying (e.g., for coatings), roll‐to‐roll printing, injection molding, deep drawing, etc.

Nevertheless, the trend toward digital fabrication has been apparent for some time in order to further advance digitalization in this area enabling the processing of materials to the desired/personalized shape.^[^ ^77^ ^]^ A benchmark technology here was 3D printing.^[^ ^78^ ^]^ Producing materials in the desired 3D shape has been a technological revolution and is constantly evolving,^[^ ^79^ ^],^ for example, 3D printing of cells resulting in fully functional human hearts components.^[^ ^80^ ^]^ Additionally, there are also industrial applications known for 3D‐printining. For example, parts of the Boeing 777 are produced by 3D‐printing.^[^ ^81^ ^]^ Meanwhile, almost all classes of materials are printable, although different techniques have to be applied, for example, fused layer molding, laser beam melting, screen printing, and two‐photon‐polymerization. The latest trend in digital fabrication is so called 4D printing, which involves printing materials that change their shape over time (fourth dimension).^[^ ^82^ ^]^ A classic example of this behavior can be found for shape‐memory materials, which are then also usable for various purposes, for example, medical applications.^[^ ^83^ ^]^

In the field of digital transformation of materials research, fabrication is probably the most advanced area, but there is still enormous potential for future developments. In particular, the printing of all kinds of materials (e.g., glass^[^ ^84^ ^]^) or the printing of high‐performance materials are still at the beginning of research.^[^ ^85^ ^]^ Furthermore, new developments regarding automated approaches combined with self‐optimizing programs enable new possibilities, such as recently reported for film‐preparation of solar cell materials.^[^ ^86^ ^]^

## 7 Conclusion—The Next Generation of Materials Research (Challenge and Possibilities)

The future of material research is becoming increasingly digital. However, this process will require significant efforts and changes. Nevertheless, the first trends are already recognizable and there is progress in many areas. The different classes of materials are currently at different levels, which are also due to the fundamentally different methods of synthesis, producing, analyzing and simulating the materials.

**Figure 2:**
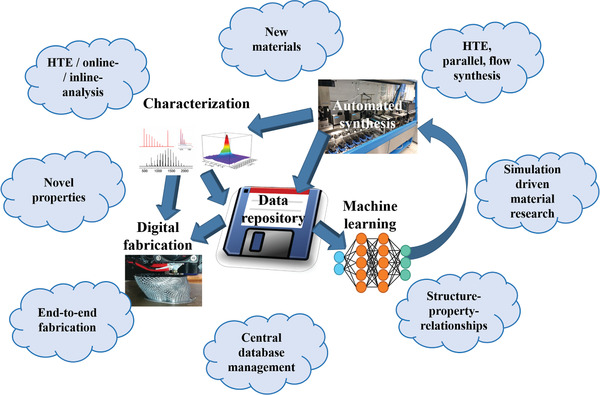
Schematic representation of a completely automated and digital‐transformed material research combining automated synthesis, online/inline characterization, machine learning, and digital fabrication. All obtained data will be stored centrally in a data repository enabling new materials as well as general structure‐property relationships.

In general, substantial progress beyond the individual sub‐areas is required in order to reach the next level. A combination of all areas—from synthesis to characterization, data management and machine learning to digital fabrication—is possible and required (**Figure** **2**). Engineering a full chain of knowledge will enable a fully automated, digital, and robot‐assisted material research and will change the way we discover, develop, and process new materials. Currently, only individual and isolated approaches are described.

As a consequence, a faster and higher reproducible development of materials will be possible. Moreover, the number of duplicates in experimentation will be reduced significantly and the human resource will have to do less synthesis work and can focus more on planning new materials and their properties in order to enable a targeted experimental design. Moreover, a higher number of experiments and a central storage of the required data will enable much more innovations resulting also in materials with properties, which are hardly imaginable up to now.

There are many challenges for achieving automated, digital, and robot‐assisted materials research (summarized in **Table** **2**). First of all, the further development of existing technologies has to be pushed forward, for example, robots and machine‐learning. Herein, the programs of AI have to be modified to more suitable for materials science and the different requirements of the different classes of materials.

**Table 2:** Summary of advantages, disadvantages as well as challenges of a more automated and digital material research

Category	Advantages	Current disadvantages	Challenges
Synthesis	– Faster – Automated – More reliable	– Expensive – Not applicable for all materials – Not accessible for all scientist – Required space for synthesis robots	– Access for all – More reaction parameters, miniaturization – Application for all materials – Purification of materials
Characterization	– Faster – Online and inline	– Reliability? – Accuracy of results	– Faster, but still reliable – Automated evaluation – Combining methods (also with synthesis)
Machine learning	– Automated optimization – Material design, which is not so easy imaginable	– Black box—hardly controllable – Loss of unexpected results – Number of iterations—time consuming experimentation	– Better programs – Data input must be ensured – Output parameters have to be defined – Establishment of simulation as data source
Digital fabrication	– Device of choice can be produced – Personalization	– Partially difficult for some materials – Not efficient for high number production	– More development, for example, 4D‐printing also for other materials – More cost‐efficient – Access to the devices
Data handling	– Central access to data – Negative results can be stored – Less duplicates of experiments – Reliable data – Meta‐analysis – Structure‐property‐relationship	– Invalid data has to be filtered out	– Development of such a system – Data format must be uniform – Information standards have to be defined – Rethinking of scientist
Teaching	– Broader teaching	– Changed profile of teacher – Partially loss of details in academia teaching	– Combination of materials science and other areas (informatics, robotics) – Implementation into curriculum

The establishment of a data repository is also crucial, as this is the only way to ensure utilization of the produced data by every scientist. Moreover, the data formats have to be defined in a standardized manner in order to have the same format across the world. Thus, all data can be reused sufficiently to evaluate general structure‐property relationships. One of the greatest challenges, however, is the training of skilled students and scientist. Classically, scientists today receive training in materials research, chemistry, engineering or physics. In the future, knowledge in the field of computer science, database management, and robotics will be required as well. Integrating this content into teaching and training represents a key challenge for the future of digital material research and will result in a new generation of scientists.

Finally, the question of the perspective and the chance for scientists remains in such a construct. What tasks will you have, and which skills do you have to bring and what can be the benefit? First, it should be noted that the realization of a digital material research is still far away and many scientists are required in order to optimize and expand the individual areas until this vision can become reality. Herein, many new discoveries are required, which must be performed by experts. In addition, properties, which should be obtained at the very end, must also be specified by a scientist and a rough direction for the starting parameters of experiments must be guaranteed. Otherwise, the number of iterations is far too large. Finally, programs need to be developed further, robots have to be optimized and made available in a cost‐efficient manner for the broad community of researchers, and the design of experiment (at least in the early stages) will remain a long‐term task for scientists. The chance of a more digital material research lies in the higher productivity, the better reliability of the data and in a better understanding of general structure‐property relationships. Scientists can focus on the major part of the discovery of new materials, which is the designing of desired materials and their properties, since the synthesis, characterization, and optimization of the process (improvement of the synthetic procedure) will be performed automatically. Consequently, faster material design and more target‐oriented research will be accessible.

## Conflict of Interest

The authors declare no conflict of interest.
